# Pim kinase inhibition sensitizes FLT3-ITD acute myeloid leukemia cells to topoisomerase 2 inhibitors through increased DNA damage and oxidative stress

**DOI:** 10.18632/oncotarget.10209

**Published:** 2016-06-21

**Authors:** Kshama A. Doshi, Rossana Trotta, Karthika Natarajan, Feyruz V. Rassool, Adriana E. Tron, Dennis Huszar, Danilo Perrotti, Maria R. Baer

**Affiliations:** ^1^ University of Maryland Greenebaum Cancer Center, University of Maryland School of Medicine, Baltimore, MD, USA; ^2^ Department of Medicine, University of Maryland School of Medicine, Baltimore, MD, USA; ^3^ Department of Microbiology and Immunology, University of Maryland School of Medicine, Baltimore, MD, USA; ^4^ Department of Radiation Oncology, University of Maryland School of Medicine, Baltimore, MD, USA; ^5^ Oncology iMED, AstraZeneca, Waltham, MA, USA; ^6^ Veterans Affairs Medical Center, Baltimore, MD, USA; ^#^ Present address: Oncology Drug Discovery Unit, Takeda Pharmaceuticals International Co., Cambridge, MA, USA

**Keywords:** acute myeloid leukemia, FLT3-ITD, Pim kinase, chemotherapy, reactive oxygen species

## Abstract

Internal tandem duplication of fms-like tyrosine kinase-3 (FLT3-ITD) is frequent (30 percent) in acute myeloid leukemia (AML), and is associated with short disease-free survival following chemotherapy. The serine threonine kinase Pim-1 is a pro-survival oncogene transcriptionally upregulated by FLT3-ITD that also promotes its signaling in a positive feedback loop. Thus inhibiting Pim-1 represents an attractive approach in targeting FLT3-ITD cells. Indeed, co-treatment with the pan-Pim kinase inhibitor AZD1208 or expression of a kinase-dead Pim-1 mutant sensitized FLT3-ITD cell lines to apoptosis triggered by chemotherapy drugs including the topoisomerase 2 inhibitors daunorubicin, etoposide and mitoxantrone, but not the nucleoside analog cytarabine. AZD1208 sensitized primary AML cells with FLT3-ITD to topoisomerase 2 inhibitors, but did not sensitize AML cells with wild-type FLT3 or remission bone marrow cells, supporting a favorable therapeutic index. Mechanistically, the enhanced apoptosis observed with AZD1208 and topoisomerase 2 inhibitor combination treatment was associated with increased DNA double-strand breaks and increased levels of reactive oxygen species (ROS), and co-treatment with the ROS scavenger N-acetyl cysteine rescued FLT3-ITD cells from AZD1208 sensitization to topoisomerase 2 inhibitors. Our data support testing of Pim kinase inhibitors with topoisomerase 2 inhibitors, but not with cytarabine, to improve treatment outcomes in AML with FLT3-ITD.

## INTRODUCTION

Acute myeloid leukemia (AML) arises in hematopoietic stem/progenitor cells and is characterized by maturation arrest at the level of myeloid blasts [1]. The class III receptor tyrosine kinase fms-like tyrosine kinase-3 (FLT3) is expressed on normal hematopoietic stem/progenitor cells and its signaling is activated by binding of FLT3 ligand [2]. FLT3 is expressed on AML cells in 70-100 percent of cases [2], and internal tandem duplication (ITD) within the FLT3 juxtamembrane domain, conferring constitutive kinase activation [3], is present in 30 percent [4, 5].

Patients with AML with FLT3-ITD have poor outcomes with current therapies. Initial treatment of AML consists of remission induction chemotherapy, including combinations of cytotoxic drugs, most commonly the nucleoside analog cytarabine (AraC) and an anthracycline topoisomerase 2 inhibitor, either daunorubicin (DNR) or idarubicin [1]. Other chemotherapy drugs active in AML include the topoisomerase 2 inhibitors mitoxantrone (MXR) and etoposide (VP-16) [1]. Post-remission therapy consists of additional chemotherapy, most commonly high-dose AraC, and/or allogeneic hematopoietic stem cell transplantation (alloSCT) [1]. However, FLT3-ITD AML patients relapse rapidly following chemotherapy [4, 5] and also following alloSCT [6]. Given the high incidence of FLT3-ITD mutations and their adverse prognostic significance, efforts have been directed toward developing targeted agents that inhibit FLT3 signaling. To date, FLT3 inhibitors used as single agents have limited and transient clinical activity [7–10]. Thus, inhibition of other key molecular targets in the FLT3-ITD pathway is currently being explored.

The Pim kinases Pim-1, Pim-2 and Pim-3 are a family of serine/threonine kinases involved in the regulation of multiple cellular functions including cell cycle, proliferation, apoptosis and drug resistance [11–15]. Pim-1 is transcriptionally upregulated by FLT3-ITD-generated signals and directly contributes to the proliferative and anti-apoptotic activity of FLT3-ITD [16]. Moreover Pim-1 phosphorylates and stabilizes FLT3-ITD, thereby promoting FLT3 signaling in a positive feedback loop [17, 18]. Thus, Pim-1 represents an attractive therapeutic target in AML with FLT3-ITD. Small molecules that inhibit Pim kinases have entered early phase clinical trials [19–22].

Our laboratory showed that concurrent treatment with the pan-Pim kinase inhibitor AZD1208 [20, 23] significantly enhances apoptosis induction by FLT3 inhibitors in cells with FLT3-ITD, in association with synergistic post-translational downregulation of the anti-apoptotic protein Mcl-1 [24]. Here, we sought to determine the effect of Pim kinase inhibition on apoptosis induction by chemotherapy drugs in cells with FLT3-ITD. We focused on chemotherapy drugs used to treat AML, including the topoisomerase 2 inhibitors DNR, MXR and VP-16 and the nucleoside analog AraC.

## RESULTS

### Pharmacologic or genetic inactivation of Pim-1 enhances the growth inhibitory and pro-apoptotic effects of topoisomerase 2 inhibitors in FLT3-ITD cell lines and primary AML cells

**Figure 1:**
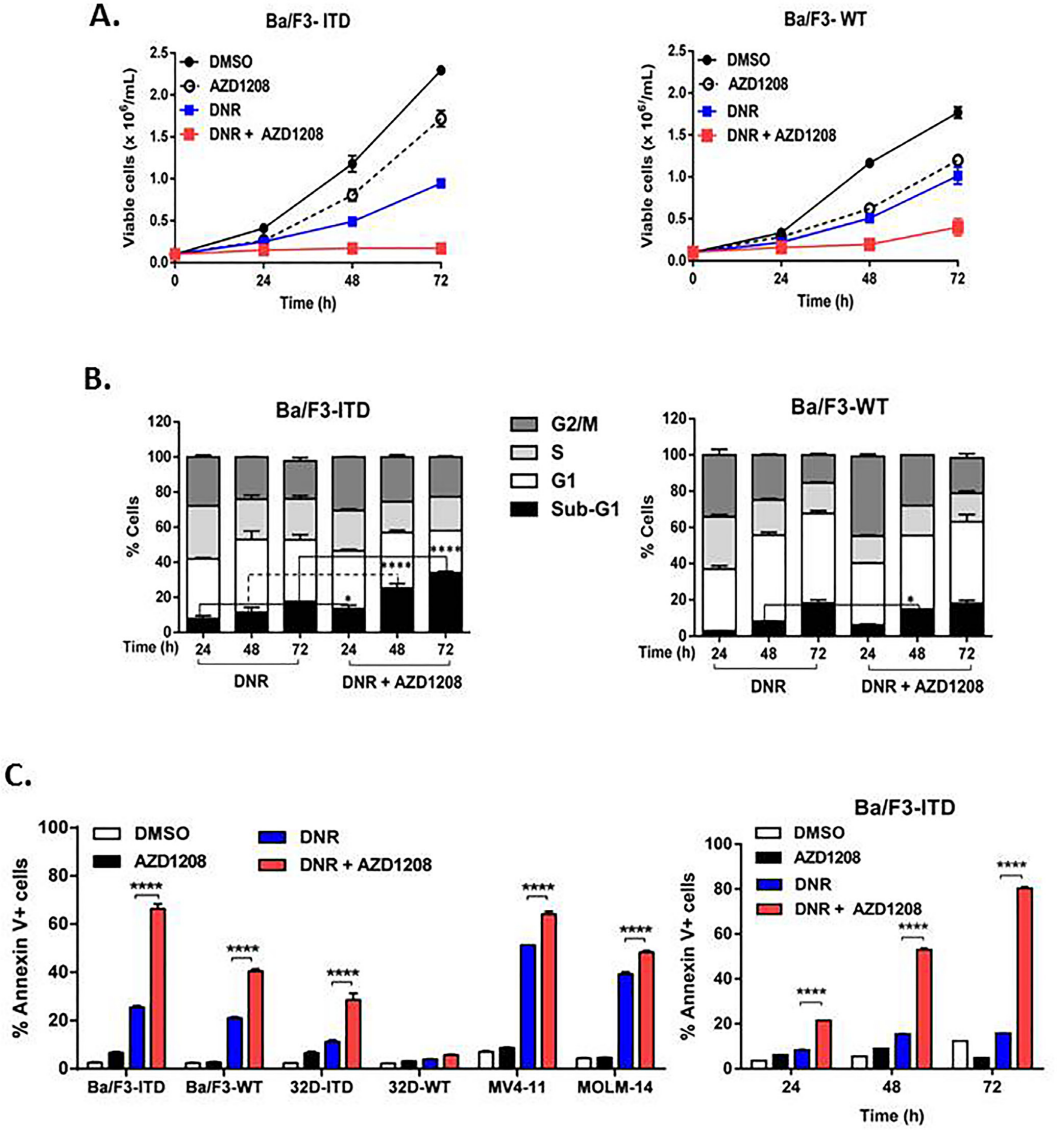
Pim kinase inhibitor sensitizes FLT3-ITD cells to apoptosis induction by topoisomerase 2 inhibitors A, B. Ba/F3-ITD and Ba/F3-WT cells were cultured with AZD1208 and/or DNR **A. AZD1208 and topoisomerase 2 inhibitor co-treatment abrogates growth of Ba/F3-ITD cells.** Viable cells were counted at 24, 48 and 72 hours. A lesser effect was seen in Ba/F3-WT cells co-treated with AZD1208 and DNR. Means ± S.E.M. of triplicate experiments are shown. **B. Sub-G1 Ba/F3-ITD cells increase with AZD1208 and topoisomerase 2 inhibitor co-treatment.** Cells were fixed and stained with propidium iodide and percentages of cells in different phases of the cell cycle were analyzed. In contrast to findings in Ba/F3-ITD cells, co-treatment with AZD1208 and DNR, relative to DNR alone, very modestly increased sub-G1 Ba/F3-WT cells. Means ± S.E.M. of triplicate experiments are shown. **C. AZD1208 and topoisomerase 2 inhibitor co-treatment increases Annexin V labeling in FLT3-ITD cells.** FLT3-ITD cells, including Ba/F3-ITD, 32D-ITD, MOLM14 and MV4-11, and FLT3-WT cells, including Ba/F3-WT and 32D-WT, were cultured with AZD1208 and/or DNR, and apoptosis was measured at 48 hours by flow cytometric analysis of Annexin V-APC and IR dye labeling. Time-dependent effects of DNR and AZD1208 are shown on the right. Means ± S.E.M. of triplicate experiments are shown. *P<0.05, ****P<0.0001.

To determine whether inhibition of Pim-1 kinase by the pan-Pim kinase inhibitor AZD1208 enhances the growth inhibitory and pro-apoptotic effects of chemotherapy drugs in FLT3-ITD AML cells, the IC_50_ concentrations of DNR, MXR, VP-16 and AraC in FLT3-ITD- and FLT3-WT-expressing cell lines were first measured using the WST colorimetric assay (Supplementary Figure S1; Supplementary Table S1). Treatment of Ba/F3-ITD cells with DNR or, to a lesser extent, MXR or VP-16 as single agents at their IC_50_ concentrations, or AZD1208 at 1 μM, resulted in a time-dependent inhibition of cell proliferation that was enhanced upon co-treatment with AZD1208 (Figure 1A, left, Supplementary Figure S2A). Moreover, AZD1208 enhancement of topoisomerase 2 inhibitor effect was seen preferentially in FLT3-ITD cells, as the effect of AZD1208 and DNR co-treatment on Ba/F3-WT cell growth was more modest (Figure 1A, right).

To determine whether the effect of concurrent treatment with Pim kinase inhibitor and topoisomerase 2 inhibitors on growth of Ba/F3-ITD cells was cytotoxic or cytostatic, effects on cell cycle and on induction of apoptosis were studied. While AZD1208 did not alter cell cycle as a single agent (Supplementary Figure S2B), it increased the cytotoxic effect of topoisomerase 2 inhibitors, as demonstrated by a significant (P<0.0001) increase in percentage of Ba/F3-ITD cells in the sub-G1 fraction (Figure 1B, left, Supplementary Figure S2B). In contrast, Ba/F3-WT cells co-treated with AZD1208 and DNR, relative to DNR alone, showed only a modest (P<0.05) increase in sub-G1 cells (Figure 1B, right). AZD1208 significantly (P<0.0001) increased topoisomerase 2 inhibitor-induced apoptosis in Ba/F3-ITD, 32D-ITD, MV4-11 and MOLM-14 cells, measured by Annexin V staining (Figure 1C, left, Supplementary Figure 3A, B, left). Increased apoptosis occurred in a time-dependent manner, with a small increase at 24 hours, followed by a progressive increase at 48 and 72 hours in Ba/F3-ITD cells co-treated with AZD1208, compared to topoisomerase 2 inhibitor alone (Figure 1C, right, Supplementary Figure S3A, S3B, right). Ba/F3-WT and 32D-WT cells were less sensitive than Ba/F3-ITD and 32D-ITD cells to apoptosis induction by AZD1208 and DNR (Figure 1C, left).

**Figure 2:**
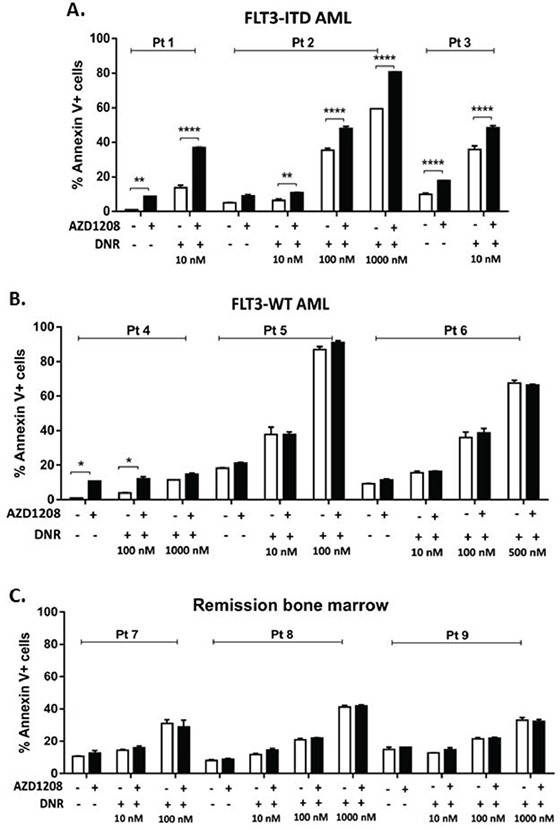
Pim kinase inhibitor sensitizes FLT3-ITD primary AML patient samples, but not FLT3-WT AML or remission bone marrow samples, to apoptosis induction by topoisomerase 2 inhibitors Mononuclear cells from bone marrow or peripheral blood of **A.** patients with AML expressing FLT3-ITD or **B.** patients with AML expressing FLT3-WT or **C.** remission bone marrow, were treated with AZD1208 and/or DNR. Apoptosis was measured at 48 hours by analysis of Annexin V-APC and IR dye labeling. *P<0.05, **P<0.005, ****P<0.0001.

The effect of combined Pim kinase inhibitor and chemotherapy drugs on primary bone marrow and blood cells from AML patients (Supplementary Table S2) was then studied. Co-treatment of FLT3-ITD-expressing primary AML cells with 1 μM AZD1208 caused a significant (P<0.01) concentration-dependent increase in apoptosis induction by topoisomerase 2 inhibitors (Figure 2A, Supplementary Figure S3A, S3B, left). In contrast, co-treatment with AZD1208 did not sensitize primary AML patient cells with FLT3-WT (Figure 2B) or remission bone marrow cells (Figure 2C) to apoptosis induction by topoisomerase 2 inhibitors. Of note, while cells from Patients 5 and 6, with FLT3-WT AML, showed close to maximum induction of apoptosis with DNR alone at 100 nM and 500 nM, respectively, lack of enhanced apoptosis in the presence of AZD1208 was seen at the lower DNR concentrations tested (Figure 2B).

**Figure 3:**
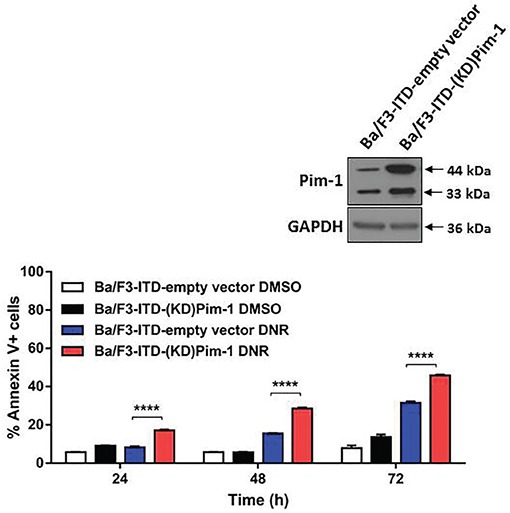
FLT3-ITD cells with kinase-dead mutant Pim-1 kinase are sensitized to apoptosis induction by topoisomerase 2 inhibitor Ba/F3-ITD cells stably expressing kinase-dead (KD) mutant Pim-1 protein or empty vector were treated with DNR. Total cell lysates were resolved by SDS-PAGE and immunoblotted with Pim-1 and GAPDH primary antibodies. Increased expression of the Pim-1 44 kDa and 33 kDa isoforms was seen in Ba/F3-ITD cells expressing KD mutant Pim-1 protein (inset). Apoptosis was measured by analysis of Annexin V-APC and IR dye labeling. Means ± S.E.M. of triplicate experiments are shown. ****P<0.0001.

To confirm that sensitization of FLT3-ITD cells to topoisomerase 2 inhibitors by AZD1208 is due to Pim kinase inhibition, rather than to off-target effects, Ba/F3-ITD cells expressing kinase-dead (KD) mutant Pim-1 [25, 26] (Figure 3, inset) were studied. DNR-induced apoptosis was significantly (P<0.0001) greater in Ba/F3-ITD cells expressing KD mutant Pim-1, compared to empty vector (Figure 3).

### Inhibition of Pim-1 kinase does not sensitize FLT3-ITD cells to AraC-induced apoptosis

**Figure 4:**
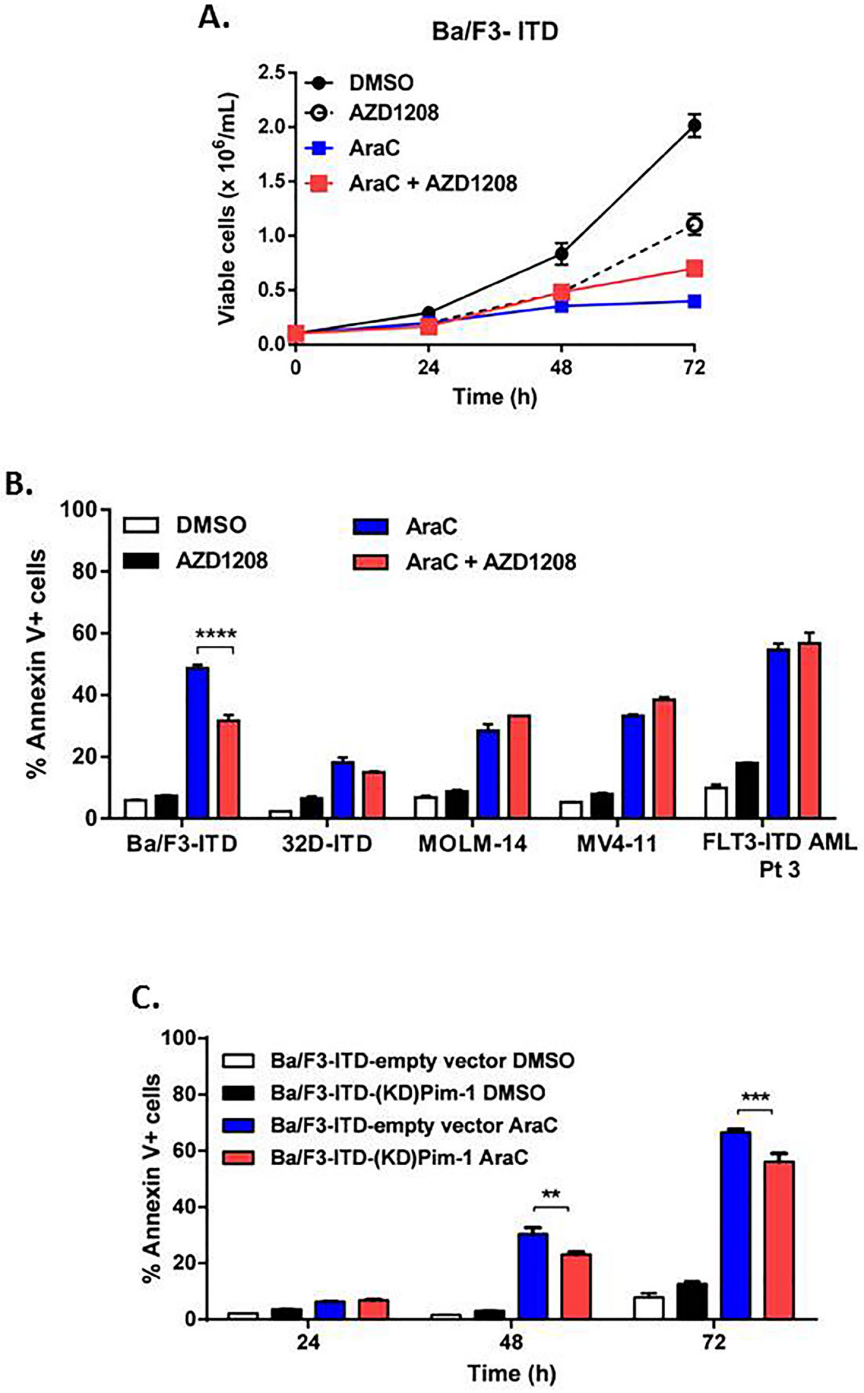
Pim kinase inhibitor does not sensitize cells expressing FLT3-ITD to apoptosis induction by AraC **A. AZD1208 does not enhance AraC suppression of Ba/F3-ITD cell growth**. Ba/F3-ITD cells were cultured with AZD1208 and/or AraC and viable cells were counted at 24, 48 and 72 hours. Means ± S.E.M. of triplicate experiments are shown. **B. AZD1208 and AraC co-treatment does not increase Annexin V labeling in FLT3-ITD cells.** FLT3-ITD cells, including Ba/F3-ITD, 32D-ITD, MOLM-14 and MV4-11 and mononuclear cells harvested from peripheral blood of a patient with AML expressing FLT3-ITD, were cultured with AZD1208 and/or AraC. Apoptosis was measured at 48 hours by analysis of Annexin V-APC and IR dye. Means ± S.E.M. of triplicate experiments are shown. **C. FLT3-ITD cells with kinase-dead mutant Pim-1 kinase are not sensitized to apoptosis induction by AraC**. Ba/F3-ITD cells stably expressing kinase-dead (KD) mutant Pim-1 protein or empty vector were treated with AraC. Apoptosis was measured by analysis of Annexin V-APC and IR dye labeling. Means ± S.E.M. of triplicate experiments are shown. **P<0.005, ***P<0.0005, ****P<0.0001.

In contrast to findings with topoisomerase 2 inhibitors, AZD1208 did not enhance the growth inhibitory activity of AraC in Ba/F3-ITD cells, and actually modestly protected cells from AraC-induced growth suppression (Figure 4A). Consistent with this lack of enhanced growth suppression, co-treatment with AZD1208 did not sensitize Ba/F3-ITD, 32D-ITD, MV4-11 or MOLM-14 cells to apoptosis induction by AraC; in fact Ba/F3-ITD were modestly protected from apoptosis induction by AraC (Figure 4B). Moreover co-treatment of FLT3-ITD-expressing primary AML cells with 1 μM AZD1208 did not increase AraC-induced apoptosis (Figure 4B). Similarly, AraC-induced apoptosis of Ba/F3-ITD cells was not enhanced in cells expressing the KD mutant Pim-1, and was actually modestly decreased, in relation to empty vector control cells (Figure 4C).

### Pim kinase inhibitor enhances apoptosis of FLT3-ITD cells by topoisomerase 2 inhibitors via intrinsic cell death signaling

**Figure 5:**
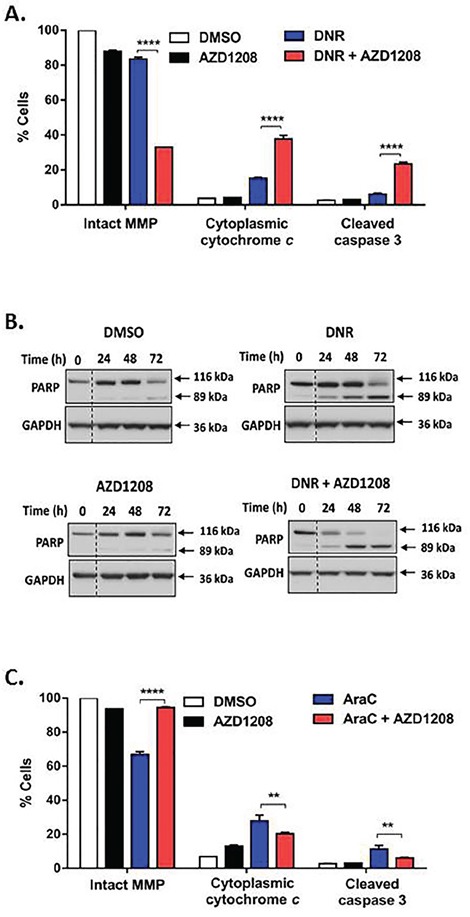
Pim kinase inhibitor and topoisomerase 2 inhibitor co-treatment increases intrinsic cell death signaling in FLT3-ITD cells **A. AZD1208 and topoisomerase 2 inhibitor co-treatment induces loss of mitochondrial membrane potential (MMP), increase in cytoplasmic cytochrome *c* and cleavage of caspase 3**. Ba/F3-ITD cells cultured with AZD1208 and/or DNR were collected at 48 hours. To measure MMP, cells were incubated with JC-1 dye and median red fluorescence was measured. To measure cytoplasmic cytochrome *c* and caspase 3 cleavage, cells were permeabilized, fixed, blocked and incubated with FITC-labeled anti-Cytochrome *c* and FITC-labeled anti-Active Caspase 3, respectively. Means ± S.E.M. of triplicate experiments are shown. **B. AZD1208 and topoisomerase 2 inhibitor co-treatment increases PARP cleavage**. Ba/F3-ITD cells were cultured with AZD1208 and/or DNR. Total cell lysates were resolved by SDS-PAGE and immunoblotted with PARP and GAPDH primary antibodies. Representative immunoblots are shown. **C. Co-treatment with AZD1208 does not increase intrinsic cell death signaling induced by AraC**. Ba/F3-ITD cells were cultured with AZD1208 and/or AraC. MMP, cytoplasmic cytochrome *c* and caspase 3 cleavage were measured as described above. Means ± S.E.M. of triplicate experiments are shown. **P<0.01, ****P<0.0001.

We then sought to determine whether enhanced apoptosis induction in FLT3-ITD cells by Pim kinase and topoisomerase 2 inhibitor co-treatment occurs via the intrinsic or the extrinsic cell death pathway. Co-treatment with AZD1208 and topoisomerase 2 inhibitors caused significantly (P<0.0001) greater decrease of mitochondrial membrane potential (MMP) (Figure 5A, Supplementary Figure S4A, S4B) and greater release of cytochrome *c* into the cytoplasm (Figure 5A, Supplementary Figure S4A, S4B), relative to topoisomerase 2 inhibitors alone, consistent with increased intrinsic cell death signaling. Additionally co-treatment with AZD1208 and DNR, compared to DNR alone, caused more pronounced cleavage of caspase 3 and its substrate PARP (Figure 5A, 5B). Increased caspase 3 cleavage was also seen in Ba/F3-ITD cells co-treated with AZD1208 and VP-16 or MXR (Supplementary Figure S4A, S4B). Moreover, enhanced caspase 3 cleavage was blocked and apoptosis was decreased by co-incubation with the pan-caspase inhibitor Z-VAD FMK (P<0.0001) (Supplementary Figure S4C), highlighting the role of caspase activation in enhanced apoptosis induction by AZD1208 and topoisomerase 2 inhibitor co-treatment.

While we previously showed that enhanced apoptosis of FLT3-ITD cells co-treated with AZD1208 and FLT3 inhibitors is associated with downregulation of the anti-apoptotic protein Mcl-1 [24], Mcl-1 levels decreased similarly in Ba/F3-ITD cells treated with DNR with and without AZD1208 (Supplementary Figure S4D), indicating that decreased Mcl-1 expression is not the mechanism for the combination effect. Expression of other pro-apoptotic and anti-apoptotic proteins, including Bad, Bim, Bax, Bak, Bcl-xl and Bcl2, also did not differ significantly (data not shown).

Additionally, co-treatment with AZD1208 did not enhance AraC-induced loss of MMP, cytochrome *c* release or caspase 3 cleavage in Ba/F3-ITD cells, and actually modestly protected from AraC-induced loss of MMP (P<0.0001) (Figure 5C) and decreased cytochrome *c* release (P<0.01) (Figure 5C) and caspase 3 cleavage (P<0.01) (Figure 5C).

### Pim kinase inhibitor enhances induction of DNA damage and ROS generation by topoisomerase 2 inhibitors, but not by AraC, in cells with FLT3-ITD

**Figure 6:**
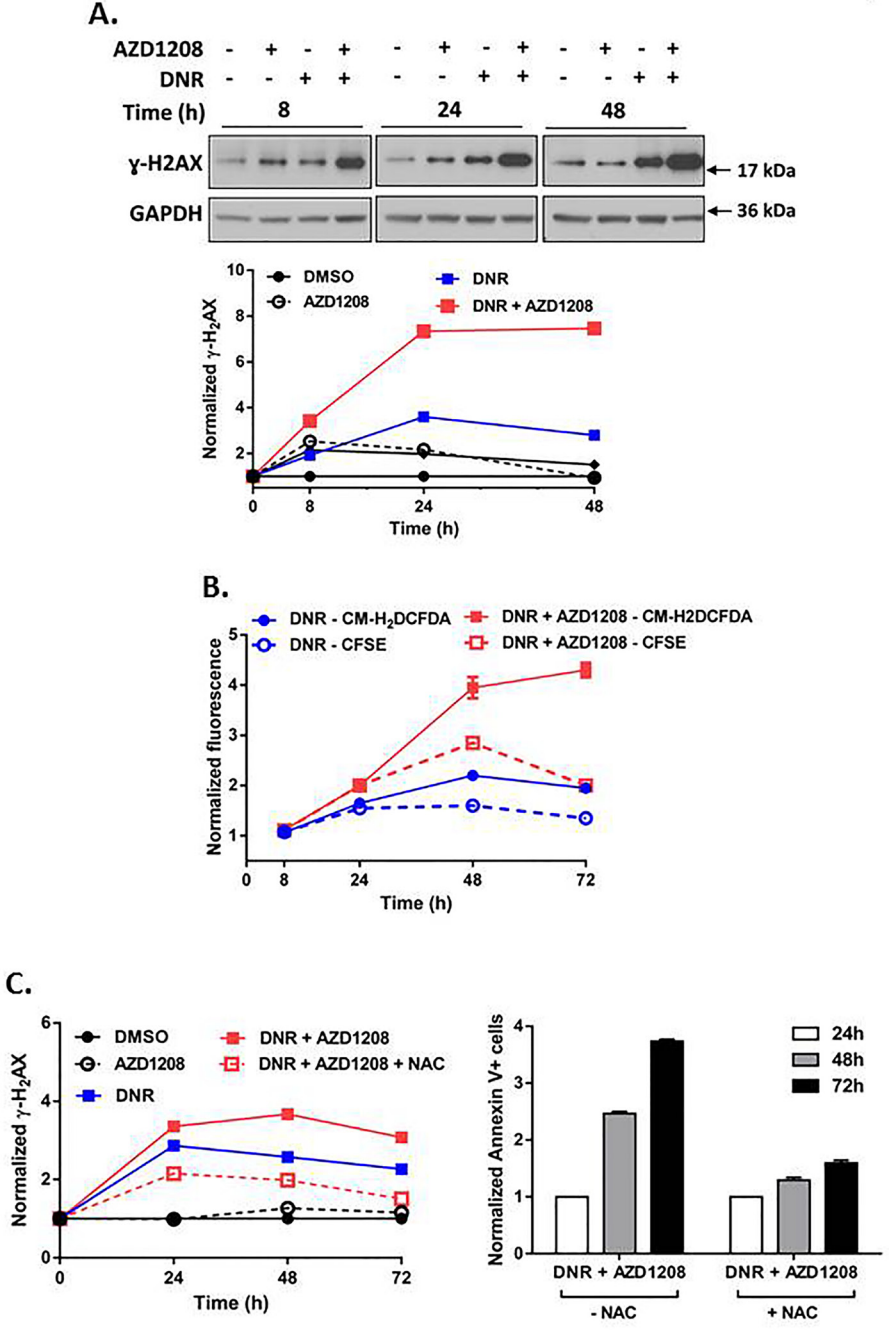
Pim kinase inhibitor enhances induction of DNA damage and ROS generation by topoisomerase 2 inhibitors in cells with FLT3-ITD **A. Concurrent treatment with Pim kinase inhibitor and topoisomerase 2 inhibitor increases DNA double-strand breaks**. Ba/F3-ITD cells were treated with AZD1208 and/or DNR. Total cell lysates were resolved by SDS-PAGE and immunoblotted with ɣ-H2AX and GAPDH primary antibodies. Densitometry was performed and intensities at serial time points were plotted relative to pre-treatment levels, defined as 1. Representative immunoblots are shown. **B. Concurrent treatment with Pim kinase inhibitor and topoisomerase 2 inhibitor increases ROS induction**. Ba/F3-ITD cells incubated with CM-H_2_DCFDA and CFSE dyes to measure intracellular ROS (solid lines) and cell proliferation (dashed lines), respectively, were treated with AZD1208 and/or DNR. Fluorescence normalized to DMSO at serial time points was plotted relative to pre-treatment levels, defined as 1. Greater CM-H_2_DCFDA fluorescence (solid lines) than CFSE fluorescence (dashed lines) indicates ROS generation. Means ± S.E.M. of triplicate experiments are shown. **C. The ROS scavenger NAC decreases DNA double-strand breaks and attenuates apoptosis induction by AZD1208 and topoisomerase 2 inhibitor co-treatment**. Ba/F3-ITD cells were treated with AZD1208 and/or DNR in the presence and absence of NAC. (left) Total cell lysates were resolved by SDS-PAGE and immunoblotted with ɣ-H2AX and GAPDH primary antibodies. Densitometry was performed and intensities at serial time points were plotted relative to pre-treatment levels, defined as 1 (left). Apoptosis was measured by flow cytometric analysis of Annexin V-APC and IR dye labeling. Fold apoptosis at serial time points was plotted relative to apoptosis at 24 hours, defined as 1. Means ± S.E.M. of triplicate experiments are shown (right).

**Figure 7:**
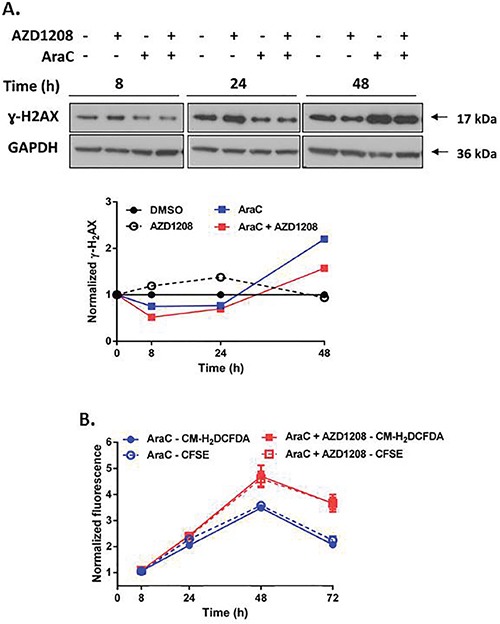
Pim kinase inhibitor does not enhance induction of DNA damage and ROS generation by AraC in cells with FLT3-ITD **A. Concurrent treatment with Pim kinase inhibitor and AraC does not increase DNA double-strand breaks**. Ba/F3-ITD cells were treated with AZD1208 and/or AraC. Total cell lysates were resolved by SDS-PAGE and immunoblotted with ɣ-H2AX and GAPDH primary antibodies. Densitometry was performed and intensities at serial time points were plotted relative to pre-treatment levels, defined as 1. Representative immunoblots are shown. **B. Concurrent treatment with Pim kinase inhibitor and AraC does not increase ROS induction**. Ba/F3-ITD cells incubated with CM-H_2_DCFDA and CFSE dyes to measure intracellular ROS (solid lines) and cell proliferation (dashed lines), respectively, were treated with AZD1208 and/or AraC. Fluorescence normalized to DMSO at serial time points was plotted relative to pre-treatment levels, defined as 1. Overlap of CM-H_2_DCFDA fluorescence with CFSE fluorescence indicates lack of ROS generation. Means ± S.E.M. of triplicate experiments are shown.

Topoisomerase 2 inhibitors stabilize the topoisomerase 2 enzyme during DNA replication, thereby causing collapse of the replication fork, which results in DNA double-strand breaks (DSBs) and subsequent cell death [27, 28]. Phosphorylated histone H2AX (γ-H_2_AX), a marker for DNA DSBs [29, 30], increased more than two-fold within 8 hours of concurrent treatment of Ba/F3-ITD cells with AZD1208 and topoisomerase 2 inhibitors, relative to topoisomerase 2 inhibitors alone, with subsequent sustained increase (Figure 6A, Supplementary Figure S5A, S5B). DNA damage induces oxidative stress, which leads to further DNA damage, creating a positive feedback loop that triggers cell death [31, 32]. AZD1208 and topoisomerase 2 inhibitor combination treatment caused minimal induction of ROS in Ba/F3-ITD cells up to 24 hours, followed by a two-fold increase at later time points, relative to treatment with topoisomerase 2 inhibitors alone (Figure 6B, Supplementary Figure S6A). Pretreatment of Ba/F3-ITD cells with the ROS scavenger NAC reduced ROS induction (Supplementary Figure S6B), as expected, and decreased induction of γ-H_2_AX expression by the combination treatment (Figure 6C, left). Moreover, apoptosis was markedly attenuated when Ba/F3-ITD cells were treated with NAC before AZD1208 and topoisomerase 2 inhibitor combination treatment (P<0.001) (Figure 6C, right, Supplementary Figure S6C). Finally, the lack of potentiation of AraC-induced apoptosis by Pim kinase inhibition reflects reduced AraC-mediated DNA damage (Figure 7A) and lack of increased ROS generation (Figure 7B) with concurrent AZD1208 treatment.

## DISCUSSION

Patients with AML with FLT3-ITD generally achieve remission following induction chemotherapy, but they have a high relapse rate and short disease-free survival [3, 4, 6]. As responses to FLT3 kinase inhibitors used as single agents are incomplete and transient, [7–10] additional targets in the FLT3-ITD signaling pathway are being explored as a basis for novel therapies. Because of the central role of Pim-1 in FLT3-ITD signaling [17, 18], and the ability of Pim kinase inhibitors to sensitize FLT3-ITD AML cells to FLT3 inhibitor-induced apoptosis [17, 18, 24, 33], we investigated the effects of Pim kinase inhibition on apoptosis induction by chemotherapy drugs in FLT3-ITD AML cells. We focused on chemotherapy drugs used to treat AML, including the topoisomerase 2 inhibitors DNR, VP-16 and MXR and the nucleoside analog AraC [1]. We used Ba/F3 as our main cell system to evaluate the differential effect of inhibiting Pim kinase in cells expressing FLT3-ITD (Ba/F3-ITD) vs FLT3-WT (Ba/F3-WT), and subsequently studied another paired set of cell lines, 32D-ITD and 32D-WT, human cell lines and, importantly, primary AML cells. Ba/F3-WT and 32D-WT cells were cultured with interleukin-3 (IL-3) due to their cytokine dependence, while Ba/F3-ITD and 32D-ITD cells are cytokine-independent [34, 35]. Ba/F3-ITD cells were also studied in the presence of IL-3, with no effect on apoptosis induction by topoisomerase 2 inhibitor and AZD1208 co-treatment (data not shown). Primary FLT3-ITD and FLT3-WT AML cells and remission bone marrow cells were cultured under similar conditions, with FBS only, without additional cytokine supplementation.

While the Pim kinase inhibitor AZD1208 had minimal cytotoxic effects as a single agent, consistent with findings in previous work [23, 24], when combined with topoisomerase 2 inhibitors it increased intrinsic apoptosis signaling in FLT3-ITD cells, leading to enhanced apoptosis. In contrast, AZD1208 did not sensitize FLT3-ITD cells to induction of apoptosis by AraC. Sensitization to topoisomerase 2 inhibitors, but not to AraC, was also seen in Ba/F3-ITD cells expressing kinase dead mutant Pim-1, confirming Pim kinase inhibition, rather than off-target effects, as the mechanism for the effect of AZD1208 on topoisomerase 2 inhibitor-induced apoptosis. Sensitization to topoisomerase 2 inhibitors was also seen in FLT3-ITD AML patient samples, but not in FLT3-WT AML patient samples or AML remission bone marrow samples. Lack of sensitization of AML remission bone marrow samples suggests a favorable therapeutic index relative to normal hematopoietic cells.

Pim kinases promote survival of cancer cells through effects on multiple cellular processes, including proliferation, cell cycle and apoptosis [11–15]. The likely existence of compensatory mechanisms among the three isoforms of Pim kinase, Pim-1, Pim-2 and Pim-3, supports the utility of developing pan-Pim kinase inhibitors [36]. Four Pim kinase inhibitors have entered clinical trials to date. SGI-1776 [19], which inhibits both Pim kinase and FLT3, showed clinical activity in phase I testing in AML, but is no longer in development due to excessive QT prolongation [37]. AZD1208, the ATP-competitive pan-Pim kinase inhibitor used in our work here, had clinical activity at well tolerated doses in phase I clinical testing in AML [20] but clinical development was discontinued due to highly variable pharmacokinetics and time-dependent decrease in exposure (personal communication). PIM447, previously LGH447 [21], and INCB53914 [22], both pan-Pim kinase inhibitors, are currently in clinical trials. We have subsequently also demonstrated enhanced apoptosis of FLT3-ITD cells treated with PIM447 in combination with topoisomerase inhibitors [38].

Pim kinase inhibition sensitizes FLT3-ITD cell lines and primary AML cells to induction of apoptosis by topoisomerase 2 inhibitors through enhanced DNA damage and increased oxidative stress. Increase in DNA DSBs was seen at 8 hours of co-treatment in the absence of increased ROS, followed by progressive induction of ROS and further DNA DSBs. Based on the kinetics of DNA DSB and ROS induction, the initial small increase in apoptosis at 24 hours in FLT3-ITD cells co-treated with AZD1208 and topoisomerase 2 inhibitors is attributable to increased DNA DSBs, which then induce oxidative stress and further DNA damage, causing the subsequent marked progressive increase in apoptosis. Protection of Ba/F3-ITD cells treated with the ROS scavenger NAC from increased apoptosis induction by combined Pim and topoisomerase 2 inhibitor treatment proves the key role of ROS induction in enhanced apoptosis.

Increased oxidative stress plays a key role in apoptosis induction by many chemotherapy drugs and small molecules [39–44]. In particular, the topoisomerase 2 inhibitors studied here are known to induce oxidative stress [45, 46]. Cellular DNA damage also increases oxidative stress [47], mediated at least in part by the p53 or H2AX/Nox1/Rac1 pathway [48]. Increased oxidative stress in turn oxidizes DNA base pairs, inducing DNA strand breaks and thereby creating a positive feedback loop [31, 32] leading to greater oxidative stress. Moreover, Pim kinase triple-knockout cells were recently reported to have increased levels of ROS, sensitizing them to K-Ras-induced cell killing [49].

The role of ROS in FLT3-ITD AML is complex. FLT3-ITD cells have high baseline ROS levels [50] which are associated with increased DNA damage. DNA damage is repaired by the error-prone alternative non-homologous end-joining (alt-NHEJ) pathway, resulting in genomic instability and disease progression [50, 51]. High levels of ROS in FLT3-ITD cells also inactivate the protein tyrosine phosphatase DEP-1/PTPRJ through oxidation of the DEP-1 catalytic cysteine, and DEP-1/PTPRJ inactivation contributes to cell transformation by FLT3-ITD [52]. On the other hand, we have shown that inhibition of Pim kinases sensitizes cells with FLT3-ITD to apoptosis induction by topoisomerase 2 inhibitors through increased ROS generation, in that marked increase in ROS is seen and enhanced apoptosis induction is inhibited by co-incubation with the ROS scavenger NAC. We hypothesize that increased baseline ROS levels in FLT3-ITD cells may sensitize them to ROS-dependent enhanced apoptosis by Pim kinase inhibitor and topoisomerase 2 inhibitor co-treatment. Recent work by several groups has focused on susceptibility of AML cells [53] and AML stem cells [53, 54] to oxidative stress, but this work has not focused on AML with FLT3-ITD in particular.

Pim kinase inhibition might also increase the DNA DSBs induced by topoisomerase 2 inhibitors by decreasing DNA DSB repair. Topoisomerase 2 inhibitors induce DNA damage by stabilizing the topoisomerase 2:DNA complex, which causes disruption of the replication fork, leading to DNA DSBs [27, 28]. DNA DSBs induced by topoisomerase 2 inhibitors are repaired by the homologous recombination (HR) and classical NHEJ pathways [55, 56]. Doxorubicin-induced DNA damage was significantly increased in lymphoma cell lines exposed to a Pim kinase inhibitor, in association with significant downregulation of the DNA DSB response proteins H2AX, ATM and Chk2 [57]. Similarly, inhibition of Pim kinase in T-cell lymphoma cells downregulated genes involved in DNA repair, including *XRCC2* (HR)*, XRCC5* (encoding Ku80, in the NHEJ pathway), and *ERCC8* (nucleotide excision repair) [36]. Pim kinase inhibition was also found to potentiate paclitaxel-induced apoptosis through decreased repair via the NHEJ pathway, with inhibition of ATM, DNA-PKcs and Ku activity and/or expression [58]. Notably, cells with FLT3-ITD exhibit downregulation of classical NHEJ, and upregulation of the alt-NHEJ pathway, which is error-prone [50, 51]. The role of Pim-1 kinase in alt-NHEJ in FLT3-ITD cells is unknown. Further studies will address whether co-treatment with Pim kinase inhibitor decreases repair of DNA DSBs induced by topoisomerase 2 inhibitors, and identify the relevant repair pathways.

Potentiation of topoisomerase 2 inhibitor-induced apoptosis by Pim inhibition was much greater in cells with FLT3-ITD than in cells with FLT3-WT. Upregulated Pim-1 expression [16], higher baseline ROS levels [50] and increased genomic instability in FLT3-ITD cells relative to FLT3-WT cells [51] likely explain this difference in sensitivity. Moreover lack of sensitization of remission bone marrow cells may predict that concurrent treatment with Pim kinase and topoisomerase 2 inhibitors in the clinical setting will be associated with a favorable therapeutic index for FLT3-ITD AML cells in relation to normal hematopoietic cells.

Pim kinase inhibition did not sensitize cells with FLT3-ITD to AraC, and actually modestly decreased induction of apoptosis by AraC. The lack of sensitization correlated with reduced DNA DSBs and lack of induction of ROS in FLT3-ITD cells co-treated with the Pim inhibitor and AraC. This is likely due to slower proliferation of FLT3-ITD cells in response to Pim inhibition, which in turn decreases the fraction of cells in S-phase, thereby decreasing the efficacy of AraC, which must be incorporated into DNA in S-phase.

These preclinical studies suggest that concurrent treatment with a Pim kinase inhibitor has the potential to sensitize AML cells with FLT3-ITD to the cytotoxic effects of topoisomerase 2 inhibitors, but not AraC. We have previously also demonstrated enhanced apoptosis of FLT3-ITD AML cells concurrently treated with Pim kinase inhibitors and FLT3 inhibitors [17, 24]. Together, our data support the potential efficacy of combining Pim kinase inhibitors with topoisomerase-2 inhibitors and with FLT3 inhibitors, likely administered sequentially, in novel treatment regimens for FLT3-ITD AML.

## MATERIALS AND METHODS

### Cell lines

FLT3-ITD- and FLT3-WT-transfected BaF3 cells (Ba/F3-ITD and Ba/F3-WT; kind gift of Dr. M. Levis, Johns Hopkins University School of Medicine, Baltimore, MD) [59] and 32Dcl3 cells (32D-ITD and 32D-WT) [50] and MV4-11 and MOLM-14 human AML cells with FLT3-ITD [60] were maintained in RPMI 1640 with 10 percent fetal bovine serum (FBS). Ba/F3-WT and 32D-WT cell cultures were supplemented with 10 ng/ml IL-3, due to the cytokine dependence of these cells [34, 35].

### Infection of Ba/F3-ITD cells with kinase-dead mutant Pim-1

Ba/F3-ITD cells were infected with pMX-Flag-K67M kinase-dead (KD)Pim-1 and empty pMX retroviral vectors, as previously described [25, 26]. Cells were collected 24 hours after infection and cultured for 96 hours with puromycin for selection. Increased Pim-1 protein expression in cells infected with (KD)Pim-1 was confirmed by immunoblotting.

### Patient samples

Mononuclear cells were isolated by density centrifugation over Ficoll-Paque (Sigma-Aldrich, St Louis, MO) from bone marrow or blood of 6 AML patients at diagnosis and bone marrow from 3 patients in remission on a University of Maryland Baltimore Institutional Review Board-approved protocol. Cells were studied without prior crypreservation. Primary FLT-3-ITD and FLT3-WT AML cells and remission bone marrow cells were cultured in RPMI 1640 with 10 percent FBS, without additional cytokine supplementation.

### Materials

The pan-Pim kinase inhibitor AZD1208 was provided by AstraZeneca (Waltham, MA). AZD1208 was used at 1 μM based on phase I clinical trial data [20] and inhibition of Bad phosphorylation at serine 112 as a pharmacodynamic endpoint [15]. DNR, MXR, VP-16, AraC and N-acetyl cysteine (NAC) (Sigma-Aldrich) were dissolved in phosphate buffered saline solution or DMSO (less than 0.05 percent) as per the manufacturer's recommendations.

### Cell proliferation assay

Cultured cells were collected at 24, 48 and 72 hours and live cells were counted after trypan blue dye exclusion.

### Cytotoxicity assay

Cytotoxicity was measured using the WST colorimetric assay, as previously described [61]. Briefly, 5,000 log-phase cells per well were incubated in 100 μL complete medium with chemotherapy drugs at increasing concentrations in 96-well plates for 48 hours. The tetrazolium salt WST-1 (Roche Diagnostics, Indianapolis, IN) was then added, cells were incubated for 2 to 4 hours and metabolically active cells were measured spectrophotometrically at 450 nM. IC_50_ values were determined by non-linear curve fitting to dose-response curves, using Prism V (GraphPad, La Jolla, CA).

### Cell cycle analysis

Cells collected at 24, 48 and 72 hours were fixed in ethanol, treated with DNase-free RNase, stained with propidium iodide (PI) for 15 minutes and analyzed on a FACSCanto II flow cytometer (BD Biosciences, San Jose, CA). Percentages of cells in different cell cycle phases were determined using FlowJo software (Tree Star, Inc., Ashland, OR).

### Measurement of apoptosis by Annexin V staining

Apoptosis was measured by flow cytometric detection of Annexin V-FITC and PI (experiments with MXR and VP-16) or Annexin V-APC and IR dye (experiments with DNR and AraC), as previously described [61].

### Measurement of mitochondrial membrane potential

Mitochondrial membrane potential (MMP) was measured using the MitoProbe™ JC-1 Assay Kit (Life Technologies, Grand Island, NY). Briefly, cells were incubated with JC-1 dye at 37°C for 30 minutes, and median red fluorescence was measured on a FACSCanto II and analyzed using FlowJo.

### Measurement of cytoplasmic cytochrome *c*

Cytoplasmic cytochrome *c* was measured with the FlowCellect™ Cytochrome *c* kit (EMD Millipore, Billerica, MA). Briefly, cells were permeabilized, fixed, blocked and incubated with anti-Cytochrome *c-*FITC for 30 minutes. Samples were acquired on a FACSCanto II and analyzed using FlowJo.

### Measurement of caspase 3 activation

Cleaved caspase 3 was measured with the FITC Active Caspase 3 Apoptosis Kit (BD Pharmingen, San Jose, CA). Briefly, cells were permeabilized, fixed and incubated with FITC-labeled anti-Active Caspase 3 for 30 minutes. Samples were acquired on a FACSCanto II and analyzed using FlowJo.

### Immunoblotting

Cells were lysed in RIPA buffer (Sigma-Aldrich) with protease and phosphatase inhibitors (Roche Applied Science, Indianapolis, IN) and whole cell lysates were subjected to immunoblotting as previously described [61]. Primary antibodies to poly (ADP-ribose) polymerase (PARP), Mcl-1, Pim-1 (Cell Signaling Technology, Danvers, MA), γH2AX (EMD Milipore) and glyceraldehyde 3-phosphate dehydrogenase (GAPDH) (Calbiochem, San Diego, CA) were used. Densitometry was performed with VisionWorks LS Image Acquisition and Analysis Software (UVP, Upland, CA).

### Measurement of reactive oxygen species

Reactive oxygen species (ROS) were measured using the redox-sensitive dye CM-H_2_DCFDA (Invitrogen, Grand Island, NY). To control for altered dye retention due to cell division, another aliquot of cells was simultaneously labeled with carboxyfluorescein succinimidyl ester (CFSE) dye (eBioscience, San Diego, CA) [62]. Briefly, cells were labeled with CFSE for 24 hours or with CM-H_2_DCFDA for 30 minutes and treated with chemotherapy drug and/or AZD1208 or DMSO control. Cells were harvested at 8, 24, 48 and 72 hours and fluorescence was measured on a FACSCanto II and analyzed using FlowJo. Divergence in the CFSE and CM-H_2_DCFDA curves at serial time points indicates altered ROS generation.

### Statistical analysis

Statistical analysis was performed by two-way ANOVA with *post hoc* Bonferroni testing, using GraphPad Prism V.

## SUPPLEMENTARY FIGURES AND TABLES


