# Supplementary material for: Pim kinase inhibition sensitizes FLT3-ITD acute myeloid leukemia cells to topoisomerase 2 inhibitors through increased DNA damage and oxidative stress
Source: Oncotarget. 2016 Jun 21;7(30):48280–95. doi: 10.18632/oncotarget.10209 (PMC5217017; doi:10.18632/oncotarget.10209)
Supplement: Supplementary file 1 [file oncotarget-07-48280-s001.pdf]

Pim kinase inhibition sensitizes FLT3-ITD acute myeloid leukemia cells to topoisomerase 2 inhibitors through increased DNA damage and oxidative stress

SUPPLEMENTARY FIGURES AMD TABLES

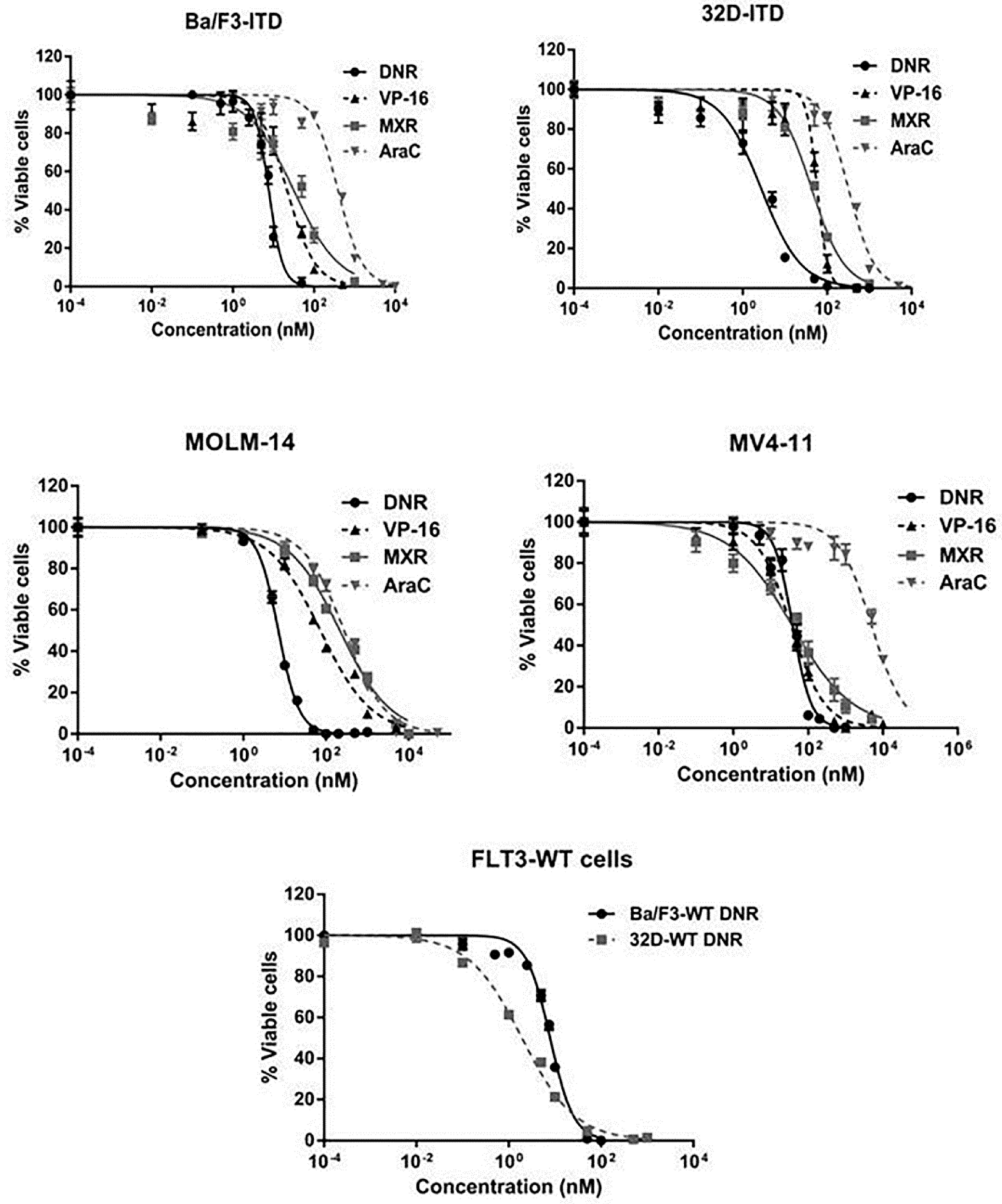

Supplementary Figure S1: Chemotherapy drug cytotoxicity. FLT3-ITD-expressing cells, including Ba/F3-ITD, 32D-ITD, MV4-11 and MOLM-14, and FLT3-WT-expressing cells, including Ba/F3-WT and 32D-WT, were incubated with topoisomerase 2 inhibitors or AraC at increasing concentrations. Absorbance was measured after addition of WST-1 reagent. IC_{50} values are shown in Table S1.

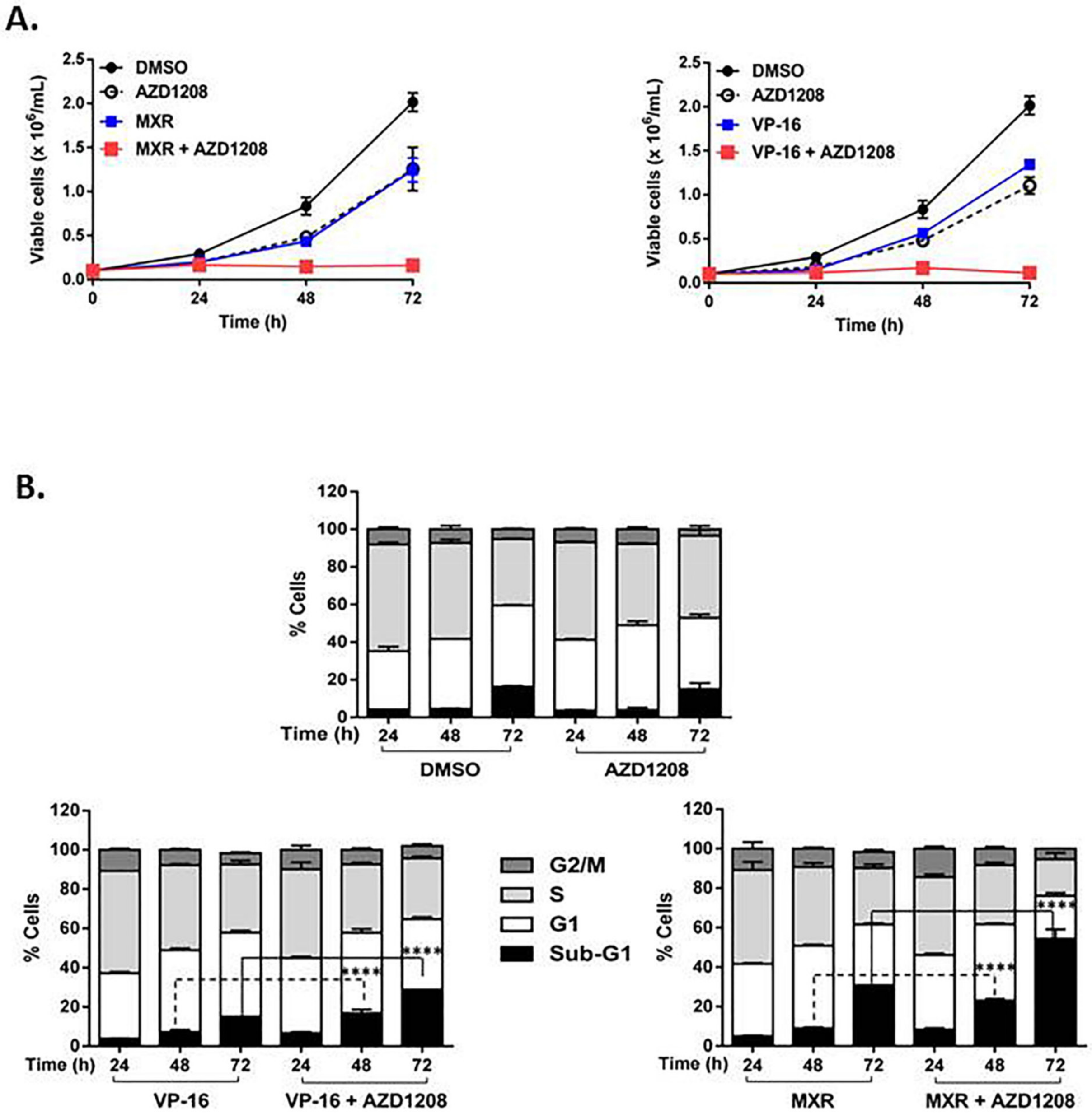

Supplementary Figure S2: Pim kinase inhibitor sensitizes FLT3-ITD cells to apoptosis induction by topoisomerase 2 inhibitors. Ba/F3-ITD cells were cultured with AZD1208 and/or VP-16 or MXR. **A. AZD1208 and topoisomerase 2 inhibitor co-treatment abrogates growth of Ba/F3-ITD cells.** Viable cells were counted as described in Figure 1A. Means ± S.E.M. of triplicate experiments are shown. **B. AZD1208 and topoisomerase 2 inhibitor co-treatment increases sub-G1 Ba/F3-ITD cells.** Percentages of cells in different phases of the cell cycle were measured as described for Figure 1B. Means ± S.E.M. of triplicate experiments are shown. *****P<0.0001*.

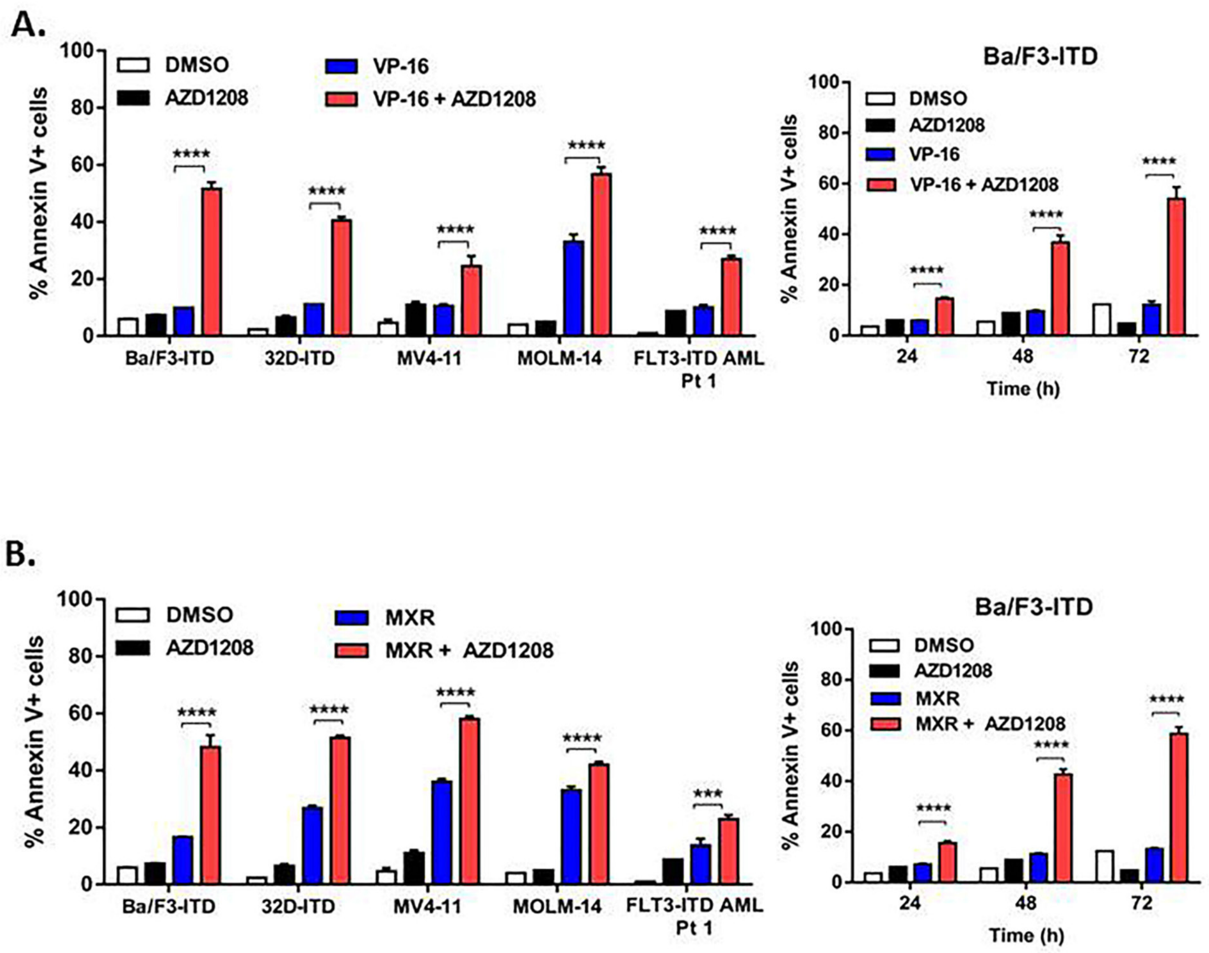

Supplementary Figure S3: Pim kinase inhibitor and topoisomerase 2 inhibitor co-treatment increases Annexin V labeling of FLT3-ITD cells. A, B. FLT3-ITD cells, FLT3-WT cells and mononuclear cells harvested from peripheral blood of a patient with AML expressing FLT3-ITD were cultured with AZD1208 and/or A. VP-16 or B. MXR., and apoptosis was measured at 48 hours by flow cytometric analysis of Annexin V-FITC and PI. Time-dependent effects of VP-16 and MXR and AZD1208 are shown on the right. Means ± S.E.M. of triplicate experiments are shown. ****$P<0.0001$.

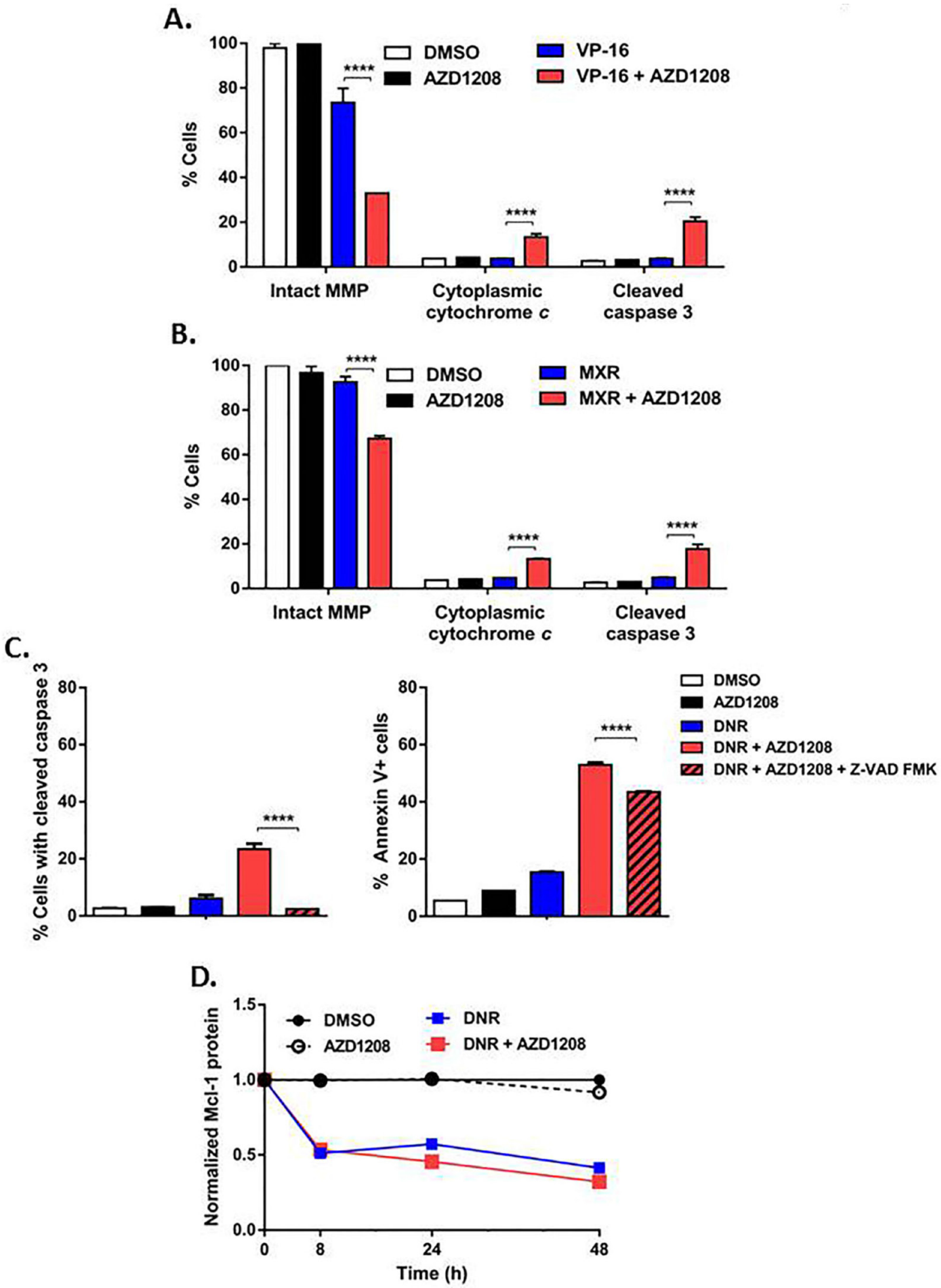

Supplementary Figure S4: Concurrent treatment with Pim kinase inhibitor and topoisomerase 2 inhibitors increases intrinsic cell death signaling in FLT3-ITD cells. A, B. AZD1208 and topoisomerase 2 inhibitor co-treatment induces loss of mitochondrial membrane potential (MMP), increase in cytoplasmic cytochrome *c* and cleavage of caspase 3. Ba/F3-ITD cells were cultured with AZD1208 and/or A. VP-16 or B. MXR. MMP, cytoplasmic cytochrome *c* and caspase 3 cleavage were measured as described in Figure 5A. Means ± S.E.M. of triplicate experiments are shown. **C. The caspase inhibitor ZVAD-FMK decreases apoptosis induction by AZD1208 and topoisomerase 2 inhibitor co-treatment.** Ba/F3-ITD cells were cultured with AZD1208 and/or DNR for 48 hours in the presence and absence of the pan-caspase inhibitor ZVAD-FMK and caspase 3 cleavage and Annexin V labeling were measured. Means ± S.E.M. of triplicate experiment are shown. **D. AZD1208 sensitization of FLT3-ITD cells to topoisomerase 2 inhibitors is not mediated by decreased Mcl-1 expression.** Ba/F3-ITD cells were cultured with AZD1208 and/or DNR. Total cell lysates were resolved by SDS-PAGE and immunoblotted with Mcl-1 and GAPDH primary antibodies. Densitometry was performed and intensities at serial time points were plotted relative to pre-treatment levels, defined as 1. ****$P<0.0001$.

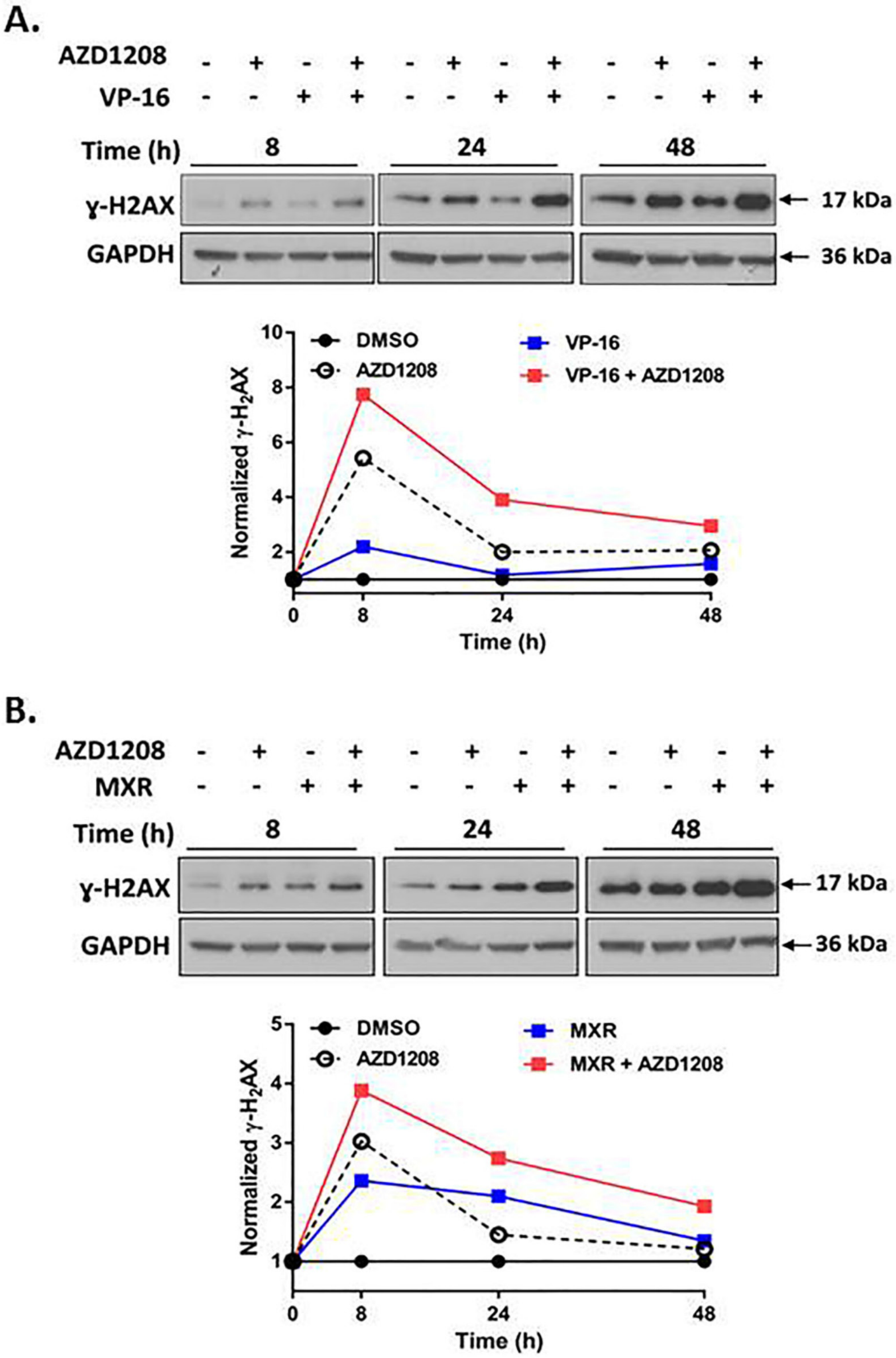

Supplementary Figure S5: Pim kinase inhibitor enhances induction of DNA damage by topoisomerase 2 inhibitors in cells with FLT3-ITD. A, B. Concurrent treatment with Pim kinase inhibitor and topoisomerase 2 inhibitors increases DNA double-strand breaks. Ba/F3-ITD cells were treated with AZD1208 and/or A. VP-16 or B. MXR. DNA DSBs were measured at serial time points as described in Figure 6A. Representative immunoblots are shown.

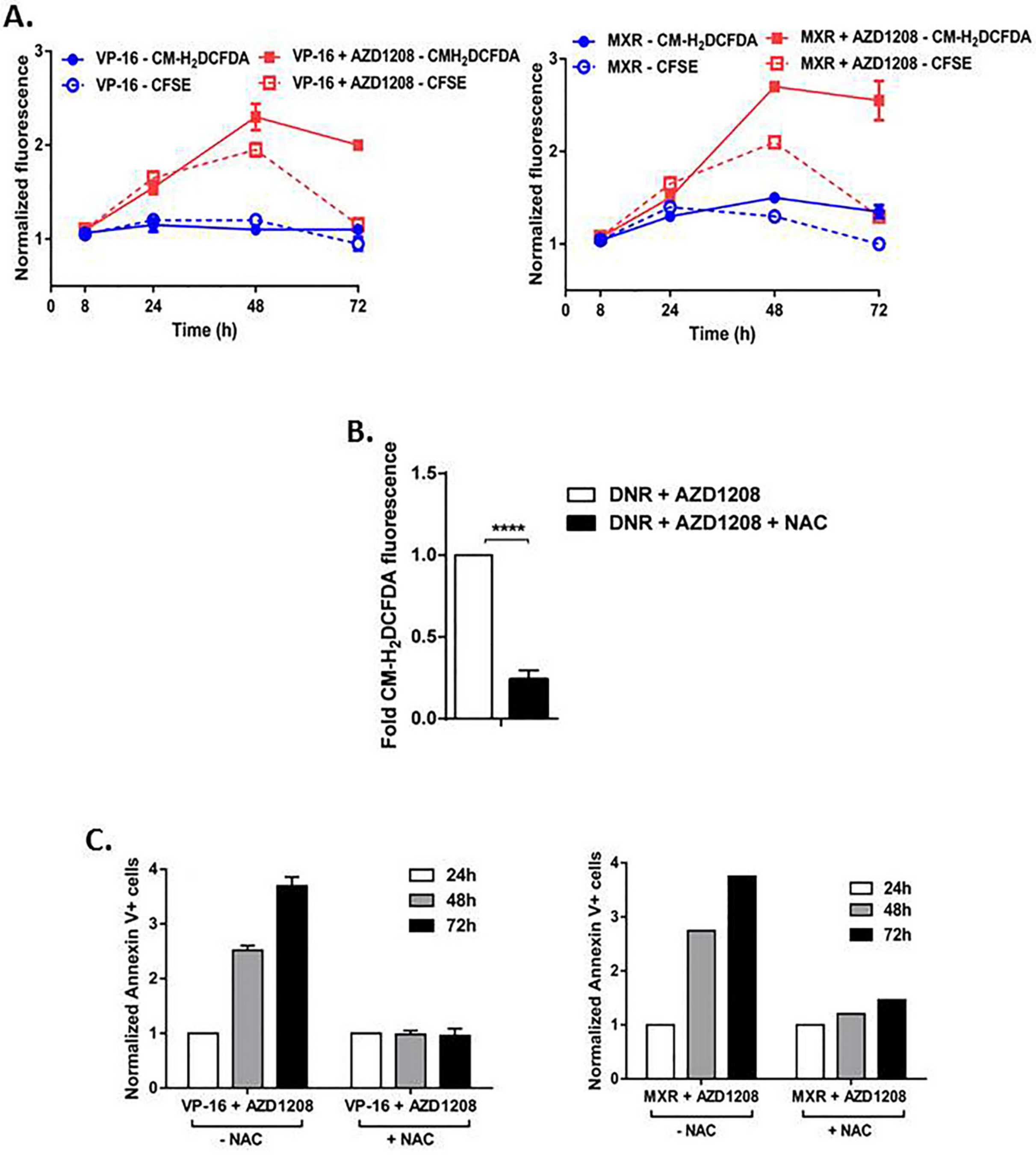

Supplementary Figure S6: Pim kinase inhibitor sensitization of FLT3-ITD cells to topoisomerase 2 inhibitors is mediated by increased induction of reactive oxygen species (ROS). A. Concurrent treatment with Pim kinase inhibitor and topoisomerase 2 inhibitors increases ROS generation. Ba/F3-ITD cells incubated with CM-H_2DCFDA (solid lines) or CFSE (dashed lines), were treated with AZD1208 and/or VP-16 or MXR. ROS generation at serial time points was measured as described in Figure 6B. Means ± S.E.M. of triplicate experiments are shown. **B. NAC pre-treatment reduces ROS induction by Pim inhibitor and topoisomerase 2 inhibitor co-treatment.** Ba/F3-ITD cells pre-incubated with CM-H_2DCFDA dye were treated with AZD1208 and DNR in the presence and absence of NAC and intracellular ROS were measured. Fluorescence in the presence, relative to absence, of NAC, defined as 1, was plotted. Means ± S.E.M. of triplicate experiments are shown. **C. Apoptosis induction by co-treatment with AZD1208 and topoisomerase 2 inhibitors is attenuated by the ROS scavenger NAC.** Ba/F3-ITD cells were treated with AZD1208 and/or VP-16 or MXR in the presence and absence of NAC. Apoptosis was measured by flow cytometric analysis of Annexin V-FITC and PI. Fold apoptosis at serial time points was plotted relative to 24 hours, defined as 1. Means ± S.E.M. of triplicate experiments are shown. ****$P<0.0001$.

Supplementary Table S1: Chemotherapy drug IC_{50} values

Cell line	FLT3	IC_{50}(nM)			
		DNR	VP-16	MXR	AraC
**Ba/F3-ITD**	ITD	7.7	21.9	32	392.4
**Ba/F3-WT**	WT	8.5	ND	ND	ND
**32D-ITD**	ITD	2.8	58.4	43.2	321.8
**32D-WT**	WT	2	ND	ND	ND
**MOLM-14**	ITD	7.1	75	220	275.7
**MV4-11**	ITD	38.3	34.3	36.3	4900

IC_{50}: Inhibitory concentration 50; DNR: Daunorubicin, VP-16: Etoposide,
MXR: Mitoxantrone, AraC: Cytarabine.

Supplementary Table S2: Clinical characteristics of AML patients whose samples were studied

Patient	Sample	Time point	Age/ sex	Karyotype	FLT3-ITD allelic burden (%)
**FLT3-ITD**					
1	PB	Diagnosis	61F	44-47,X,-X,add(1)(p36.1),add(9)(q34), +13,+1-2mar	62
2	BM	Diagnosis	20F	46,XX	61
3	PB	Diagnosis	41F	46,XX	8
**FLT3-WT**					
4	PB	Diagnosis	78F	48,XX,+2mar	N/A
5	BM	Diagnosis	43F	48-50,XX,+2,t(6;10)(p21;q26),t(11;19)(q23;p13.3),+20,+21	N/A
6	BM	Diagnosis	78M	47,XY,+8	N/A
**Remission**					
7	BM	Remission	52M	46,XY	N/A
8	BM	Remission	38M	46,XY	N/A
9	BM	Remission	67F	46,XX	N/A

PB: Peripheral blood; BM: Bone marrow; M: Male; F: Female; N/A: Not applicable.
